# Temporomandibular signs, symptoms, joint alterations and disease activity in juvenile idiopathic arthritis – an observational study

**DOI:** 10.1186/1546-0096-11-37

**Published:** 2013-10-17

**Authors:** Anna-Lena Cedströmer, Anna Andlin-Sobocki, Lillemor Berntson, Britt Hedenberg-Magnusson, Lars Dahlström

**Affiliations:** 1 Department of Behavioral and Community Dentistry, University of Gothenburg, Institute of Odontology at the Sahlgrenska Academy, Box 450, SE-405 30 Göteborg, Sweden; 2 Department of Orthodontics, Eastman Institutet, Folktandvården Stockholms län AB, Stockholm, Sweden; 3 Department of Surgical Sciences, Oral and Maxillofacial Surgery, Uppsala University Hospital, Uppsala, Sweden; 4 Department of Women’s and Children’s Health, Uppsala University, Uppsala, Sweden; 5 Department of Dental Medicine, Section for Orofacial Pain and Jaw Function, Karolinska Institutet, Huddinge, Sweden; 6 Department of Clinical Oral Physiology, Eastman Institutet, Folktandvården Stockholms län AB, Stockholm, Sweden

**Keywords:** Adolescent, Arthritis juvenile rheumatoid/diagnoses, Child, Retrospective studies, Temporomandibular joint

## Abstract

**Background:**

Juvenile idiopathic arthritis (JIA) is a heterogeneous disease that frequently affects also the temporomandibular joint (TMJ) and associated structures. The main aim of this observational study was to describe systematically orofacial clinical signs and subjective symptoms in JIA patients, classified according to the International League of Associations for Rheumatology (ILAR) criteria, and to relate the findings to disease activity and radiological TMJ condyle lesions.

**Methods:**

The study was a retrospective evaluation of dental and medical records in consecutive JIA patients referred to one of three dental specialist clinics in Sweden during an eight-year period. Data concerning temporomandibular signs, symptoms and general disease activity were collected and condylar alterations were judged on panoramic radiographs.

**Results:**

All ILAR categories of JIA were represented among the 266 referrals included in the study. The distribution of patients among categories resembled the pattern seen in epidemiological studies. Persistent oligoarthritis was the largest category with 36.5% of the patients. Temporomandibular clinical signs (mild, moderate or severe) occurred in 57.7% to 92.0%, and subjective symptoms (mild or severe) in 32.0% to 76.0% of the patients in all categories. Patients in the juvenile psoriatic arthritis category had the largest number of orofacial signs and symptoms, and patients in the persistent oligoarthritis category had the fewest signs and symptoms. There were significant associations between clinical signs as well as subjective symptoms and overall disease activity. Half of all the patients had undergone panoramic examinations and 37.9% of those were judged to have condylar alterations after a mean of 2.9 years after onset. No associations between radiological findings and variables, such as signs, symptoms or disease activity, were found.

**Conclusions:**

Temporomandibular signs and symptoms can be expected to a varying degree, including severe cases, in all JIA categories. Clinical and subjective orofacial involvement appears to be related to disease activity but not to condylar lesions.

## Background

Juvenile idiopathic arthritis (JIA) is a generic term for arthritis with unknown etiology and with the onset occurring before the age of 16 [1]. The incidence in Sweden is approximately 15 in 100 000 [2,3]. The classifications of the disease have varied over time and in different parts of the world, making comparisons between patients and disease characteristics difficult. In 1995, the new classification criteria for JIA, the so-called International League Against Rheumatism (ILAR) criteria, were proposed [1,4]. The ILAR classification is currently used worldwide based on clinical features, heredity and laboratory data.

The inflammation of the musculoskeletal system in JIA has many different clinical expressions. All joints, including the temporomandibular joint (TMJ), may be affected. Pain from the jaws during function and rest may be prominent in JIA as well as limited opening capacity, joint sounds, locking and palpatory tenderness of the TMJ and associated muscles [5]. One or both TMJs may thus be involved in JIA, sometimes without clinical or subjective expression, which may cause delay of detection [6]. The TMJ may also be the only affected joint [7]. TMJ involvement is thought to occur during the active phase of JIA, when the inflammation generates chondral and subchondral bone lesions and the consequences for mandibular growth and development may be considerable, regardless of whether or not there are signs and symptoms [8].

The prevalence of signs, symptoms and radiological findings in the masticatory system in patients with JIA has varied between 17% and 87% in previous studies [9-15]. The number of examined subjects has often been limited. The disparity may be due not only to large variations in patient sample composition but also to the examination methods used.

Clinical findings and subjective complaints in the orofacial area in young JIA patients, classified according to the now generally applied ILAR criteria, have not been extensively defined, and the relationship between orofacial engagement and disease activity and radiographic changes in the TMJ has not been determined. The main aim was therefore to describe systematically the clinical and subjective involvement of the TMJ and associated structures in pediatric patients diagnosed with JIA, categorised according to the ILAR criteria, and referred to dental specialist clinics. Another aim was to relate orofacial signs and symptoms to disease activity and to condylar alterations, as assessed on panoramic radiographs.

## Methods

All consecutive patients who fulfilled the ILAR criteria of JIA [1] and who were referred by physicians or dentists during an eight-year period to one of three specialist dental clinics in Sweden were included. The participating clinics were the Department of Surgical Sciences, Oral and Maxillofacial Surgery in Uppsala, the Orofacial Pain Specialist Clinic in Gothenburg and the Department of Clinical Oral Physiology at the Eastman Institute in Stockholm. Data were collected from the first examination by the specialist dentist (study visit), which took place between January 1, 1999, and December 31, 2006. Eligible patients had to be born after January 1, 1986, since they were covered by free dental care and therefore more likely to come to examination.

A systematic clinical assessment, according to structured protocols, of orofacial signs was made by a specialist dentist at the study visit. Anamnestic information related to patient-reported symptoms was likewise collected with standardized questions to the patient and/or parents/carers on the same occasion. One dentist (A-L C) read the evaluations by the specialist dentists retrospectively, and Helkimo’s indices [16] were calculated on the basis of the clinical and anamnestic data. Helkimo’s clinical dysfunction index, Di 0-III, evaluates mandibular mobility, TMJ function, muscle pain, TMJ pain and pain on movement of the mandible on a three-point scale of increasing severity; 0, 1 or 5. The sum, 0–25 points, constitutes the dysfunction score, which forms the basis of the clinical dysfunction index. The signs found at the clinical examination can thus be expressed as Di 0 (0 points, no signs), Di I (1–4 points, mild signs), Di II (5–9 points, moderate signs), or Di III (10–25 points, severe signs). Anamnestic data relating to subjective symptoms, the Helkimo’s anamnestic index, Ai 0-II, summarize TMJ sounds, fatigue/stiffness of the jaw, pain, difficulty of jaw movements, locking and luxation. Ai 0 denotes the complete absence of subjective symptoms and Ai I denotes mild symptoms, such as joint sounds, stiffness or fatigue of the jaws. Ai II denotes severe symptoms, with one or more of the following reported in the anamnesis: difficulty opening the mouth wide, locking, luxation or pain on movement, facial and jaw pain.

Data were also collected by the same dentist from the medical records at the pediatric rheumatology clinics where the participating patients were treated. Interpretation of all medical data was supervised by one pediatric rheumatologist (LB). The JIA patients were classified retrospectively according to the 2004 ILAR criteria [1].

The general disease activity during the last two years before the study visit was recorded. A modified version of The European League Against Rheumatism (EULAR) criteria, as presented by Andersson-Gäre [17], was used. One further category was added, yielding a modified version of five categories, where EU 1 denoted active disease (increasing number of engaged joints), EU 2 denoted stable disease (unchanged number of engaged joints), EU 3 denoted an inactive disease with treatment (no disease activity with treatment), EU 4 denoted an inactive disease without treatment (no disease activity and no treatment less than two years), and EU 5 denoted remission of the disease (no disease activity and no treatment for more than two years).

The presence or absence of any condylar alterations, morphologic (flattening, osteophyte) or structural (erosion, sclerosis, subchondral cysts), was evaluated on panoramic radiographs. The dichotomous assessment was made by one dentist (A-LC), blinded to all other information, together with an oral radiology specialist, until consensus; all on one occasion.

The multicenter study was approved by the Ethical Committee at the University of Gothenburg, Göteborg, Sweden (342–07).

### Statistics

For comparisons of Di, Ai and EU between study clinics and all eight categories, the Kruskal-Wallis test was used. In order to test the difference between every two categories, clinical and radiographic, the Mantel-Haenszel chi-square test was used. For comparison of gender and age between all sites, the Pearson chi-square test and the Kruskal-Wallis test were used, respectively. For comparison between every two categories of gender and age, Fisher’s exact test and the Mann–Whitney u-test were used, respectively. Spearman’s rank correlation was used for the correlation analysis. Two-tailed statistical analyses were performed at a significance level of P < 0.05, unless otherwise stated.

## Results

The total number of referrals during the study period was 278. Due to remission or missing medical data at the time of the first dental specialist examination, 12 of the 278 referrals could not be classified and, consequently, 266 patients were included in the study.

Twenty-six of the participants were examined in Uppsala, 53 in Gothenburg and 199 in Stockholm. One specialist dentist examined all the patients in Uppsala and one in Stockholm, while several specialists examined the patients in Gothenburg.

There were no differences among the patients between the three study clinics with regard to gender distribution, age at disease onset or at examination*.* Nor were there any differences between sites with regard to summarized signs and symptoms, Di and Ai. The dental specialist clinic in Gothenburg had more patients with extended oligoarthritis than the clinics in Stockholm and Uppsala (both P < 0.0001). The latter two sites had more patients than Gothenburg (both P < 0.0001) with persistent oligoarthritis. The Stockholm clinic also had more patients with RF-negative polyarthritis than Gothenburg (P < 0.0001)*.* Patients at the dental specialist clinic in Gothenburg had more active disease, EU 1, than the other clinics (P = 0.010). The clinic in Gothenburg performed significantly fewer radiological examinations of their patients than the clinics in Uppsala and Stockholm (both P < 0.0001).

The distribution of the 266 patients to the ILAR categories with respect to gender, mean age at disease onset and disease duration at the study visit is shown in Table 1. Among all patients at all sites, persistent oligoarthritis made up the largest category, followed by RF-negative polyarthritis.

**Table 1:** Distribution of gender, age at disease onset, and disease duration in 266 patients with JIA allocated to the ILAR categories

	**Total**	**Systemic**	**Persistent oligo**	**Extended oligo**	**RF-negative polyarthritis**	**RF-positive polyarthritis**	**Psoriatic**	**ERA**	**Other arthritis**
n (% girls)	266 (76)	5 (40)	97 (77)	38 (82)	68 (81)	15 (93)	25 (76)	5 (40)	13 (38)
Onset age, mean, 25/75^th^ percentile yrs	7.1 (3.0/10.7)	3.3 (2.1/3.9)	6.1 (2.2/9.5)	5.7 (2.6/8.1)	7.4 (3.5/10.6)	11.9 (9.9/14.4)	8.8 (5.0/12.9)	11.1 (10.1/12.5)	9.5 (7.3/11.7)
Mean disease duration at study visit, 25/75^th^ percentile yrs	2.9 (0.4/5.4)	4.8 (0.6/7.1)	2.5 (0,3/4,3)	4.2 (0.7/7.6)	2.7 (0.4/4.2)	0.8 (0.2/1.7)	3.0 (0.4/5.9)	1.2 (0.4/1.1)	3.5 (0.7/5.8)

Females dominated among all the included 266 patients, with an overall ratio of 3:1. The mean age at the first specialist dental examination (study visit) was 10.0 years (7.4/13.1 in the 25/75^th^ percentile).

**Figure 1:**
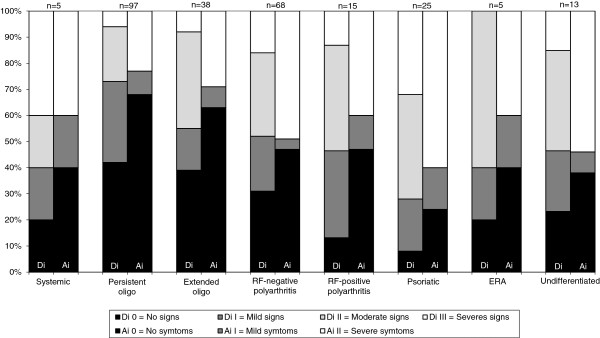
**Distribution of clinical signs, Di and symptoms, Ai.** Distribution (%) by Helkimo’s dysfunction index, Di 0-III and Helkimo’s anamnestic index, Ai 0-II, of 266 JIA patients allocated to the ILAR categories. Di 0 denotes clinically no signs, Di I mild, Di II moderate, and Di III severe signs of dysfunction of the masticatory system. Ai 0 denotes complete absence of subjective symptoms, Ai I mild, and Ai II severe symptoms of the masticatory system.

The distribution of the dysfunction index, Di, and the anamnestic index, Ai, respectively, among the patients in the ILAR categories is shown in Figure 1. Clinical signs, Di I-III (mild, moderate or severe), were found in 57.7% to 92.0% of the patients in the different ILAR categories, and subjective symptoms, Ai I-II (mild or severe), in 32.0% to 76.0% of the patients. There were significant differences between the JIA categories in both Di and Ai. Patients diagnosed with psoriatic arthritis had more clinical signs, Di, than patients with extended oligoarthritis (P = 0.0028) and RF-negative polyarthritis (P = 0.0138). Patients with psoriatic arthritis also reported more subjective symptoms, Ai, than patients with persistent oligoarthritis (P < 0.0001) and extended oligoarthritis (P = 0.040). Clinical signs, Di, were less common in the persistent oligoarthritis category compared with systemic arthritis (P = 0.0444)), RF-negative polyarthritis (P = 0.0076), RF-positive polyarthritis (P = 0.0180), and psoriatic arthritis (P < 0.0001). Patients with persistent oligoarthritis also reported fewer symptoms, Ai, than patients with undifferentiated arthritis (P = 0.0191) and RF-negative arthritis (P = 0.015). The psoriatic category therefore had the most orofacial signs and symptoms, according to Helkimo’s indices, while patients diagnosed with persistent oligoarthritis had the fewest.

The correlation between clinical signs, Di, and subjective symptoms, Ai, was r_s_ = 0.57 (P < 0.001).

**Figure 2:**
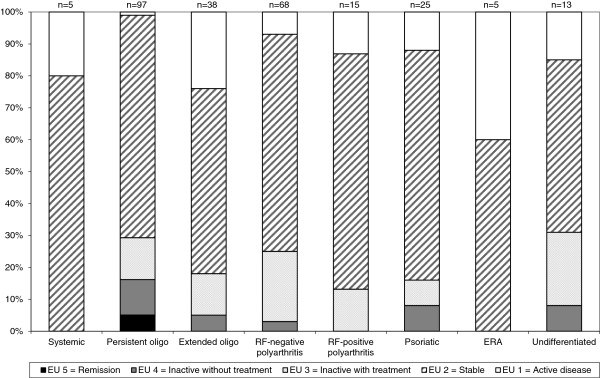
**Distribution of disease activity, EU.** Distribution (%) of disease activity according to the modified European League Against Rheumatism, EULAR (EU1-EU5) criteria, in 266 patients with JIA allocated to the ILAR categories. EU 1 denotes active disease (increasing number of engaged joints), EU 2 denotes stable disease (unchanged number of engaged joints), EU 3 denotes an inactive disease with treatment (no disease activity), EU 4 denotes an inactive disease without treatment (no disease activity and no treatment less than two years) and EU 5 denotes remission of the disease (no disease activity and no treatment for more than two years).

Among all patients, 76.0% had a disease activity of 1 or 2, Figure 2. There were significant differences between categories in disease activity, summarized for the last two years. Patients with systemic arthritis had more disease activity, EU 1, than patients with persistent oligoarthritis (P = 0.0034), extended oligoarthritis (P = 0.0201), RF-negative polyarthritis (P = 0.0395) and psoriatic arthritis (P = 0.0310). Patients with extended oligoarthritis had less disease activity than psoriatic arthritis (P = 0.0361).

Clinical signs, Di, was significantly associated with disease activity, EU, r_s_ = -0.13 (P *=* 0.0310). The association between subjective symptoms, Ai, and disease activity was also significant, r_s_ = -0.18 (P *=* 0.0044).

In half of the patient cohort, a panoramic examination was performed at the study visit (n = 134). There were no differences in the proportion of radiological examinations between categories, gender, age at onset or age at examination, Di or Ai. In patients with disease activity EU 2, radiological examinations were performed more often than in other groups (P = 0.0243). Four of the panoramic radiographs were judged as “unreadable”. Of the remaining radiographs in 130 patients, 48 (37.0%) were rated as having a condylar alteration. The distribution of Di, Ai and condylar alterations among the ILAR categories is shown in Table 2. No significant association between condylar alterations and category was observed. Nor were any significant associations between condylar alterations and gender, age at onset, age at examination, clinical signs (Di), subjective symptoms (Ai) or disease activity found.

**Table 2:** Distribution of panoramic examinations, clinical signs, Di, subjective symptoms, Ai, and condylar alterations in 130 patients with JIA allocated to the ILAR categories

	**Total**	**Systemic**	**Persistent oligo**	**Extended oligo**	**RF-negative polyarthritis**	**RF-positive polyarthritis**	**Psoriatic**	**ERA**	**Other arthritis**
Panoramic examination,n(%)	130	3 (2)	50 (39)	9 (7)	36 (28)	9 (7)	13 (10)	2 (2)	8 (6)
Di 0	45(35)	1(33)	22(44)	4(44)	12(33)	2(22)	1(8)	1(50)	2(25)
Di I	24(19)	1(33)	12(24)	0	5(14)	3(33)	2(15)	0	1(13)
Di II	44(34)	0	13(26)	3(33)	13(36)	3(33)	7(54)	1(50)	4(50)
Di III	17(13)	1(33)	3(6)	2(22)	6(17)	1(11)	3(23)	0	1(13)
Ai 0	73(56)	1(33)	34(68)	7(78)	19(53)	5(56)	3(23)	1(50)	3(38)
Ai I	11(9)	1(33)	4(8)	0	2(1)	1(11)	2(15)	0	1(13)
Ai II	46(35)	1(33)	12(24)	2(22)	15(42)	3(33)	8(62)	1(50)	4(50)
Condylar alteration, n (%)	48 (37)	0	17 (34)	7 (78)	9 (25)	6 (67)	7 (54)	0	2 (25)

## Discussion

This study collected data related to clinically recorded orofacial signs and patient-reported subjective symptoms in a comprehensive cohort of patients with JIA, referred for specialist dental examination, at a mean of 2.9 years after disease onset. ILAR categories and disease activity during the last two years were assessed. Panoramic radiographs performed at the study visit, were available for half of all patients. Records from three clinics, with geographically distinct catchment areas and with patients referred over a period of several years, were examined to ensure accuracy. Orofacial signs and symptoms were common, varied between categories, and were related to the disease activity. Condylar alterations were not associated with other variables, such as ILAR category, clinical signs, symptoms or disease activity.

A strength of the study was the fairly large cohort, with objective as well as subjective variables involved and with radiological findings as an end point. What percentage of all patients diagnosed with JIA that was referred for orofacial issues is not known. Although the cohort of referrals was relatively sizeable, in contrast with many previously described case series, the number of patients in some of the ILAR categories was small, which weakens the results. Above all, the basic data were not population-based, were collected retrospectively and only discussed in relation to previous studies. Register studies, like the present one, run the risk of selection bias with overrepresentation of severe cases possibly illustrated by the high disease activity among the referrals. Although only one dentist summarized the standardized clinical and anamnestic protocols, a group of experienced but non-calibrated examiners were involved in the original data collection. This implies a weakness, in terms of both validity and reliability.

Some differences in patient characteristics between the sites were observed, partly reflecting variations in clinical routines. The merged study cohort was representative of the ILAR categories, gender and age in a distribution resembling cohorts from epidemiological studies [3,18-20].

Different methods of collecting data regarding temporomandibular signs and symptoms have been used over the years. Helkimo’s dysfunction, Di, and anamnestic, Ai, indices are coarse quantifications of signs and symptoms in the orofacial area, but the standardized evaluations facilitate comparisons between patients and conditions. The indices have acceptable levels of reproducibility, not only for the same observer but also between different observers [16,21], and are strongly correlated with other indices [22]. Di and Ai have been widely used in epidemiological contexts concerning temporomandibular disorders (TMDs) [23], also among children and adolescents [24].

TMDs with signs and symptoms similar to those in JIA also occur in the healthy, general population. Signs and symptoms have been found to be less frequent in children than in adults in general [23]. Examination of randomly selected samples of 3, 5, 10 and 15-year-old children in a Swedish city, using Helkimo’s indices, revealed a low prevalence of severe TMD signs and symptoms [25]. Severe clinical signs, Di, were rare, also in a longitudinal series of Finnish children [26]. Signs of, at most, mild dysfunction have thus been found in only a few percent among healthy, young children [27], but increase with age [28-30]. The prevalence of self-reported TMD in a large cohort of Swedish youths, 12–19 years of age, was 4.2% [31], and other studies have reported similar figures [32,33]. Most of the patients in the present case series were afflicted in the orofacial area. Cases with severe dysfunction, Di III, were found in all but one of the diagnostic categories. Severe symptoms; Ai II, occurred in all categories. Orofacial engagement in young JIA patients, as found in this study, thus exceeds that in comparable, healthy groups. Severe signs and symptoms among JIA patients, according to Helkimo’s indices, have been presented earlier but all the categories were not represented [34].

In the present study, the prevalence of orofacial signs, as assessed by the specialist dentists, was higher than the prevalence of self-reported orofacial symptoms in all JIA categories, which is in agreement with epidemiological surveys of TMD [23]. Patients may be unaware of, used to or not disturbed by certain clinical findings. The significant correlation between signs and symptoms, as found in the present study, is supported by findings in other studies [23].

The distribution of clinical signs and reported orofacial discomfort in this cohort differed significantly between the ILAR categories. That differences exist are in line with earlier investigations but the results have varied between studies. Pedersen et al. [10] found most TMJ involvement in the polyarticular category, Twilt et al. [11] in the systemic category and Cannizzaro et al. [13] in the extended oligoarticular category. In the present study, both signs and symptoms were significantly more frequent in the psoriatic arthritis category than in several other categories, despite similar disease activity. Clinical signs were found in almost all psoriatic arthritis patients and symptoms were also common. One reason may be that psoriatic arthritis engages not only the synovium, but also the surrounding tissues [35-37].

Moreover, the RF-positive category had very few patients without any orofacial signs. It is known that the RF factor contributes to joint involvement [10,11,38,39], but as the number of patients in this category was limited, it is difficult to draw confident conclusions.

This study found significant associations between orofacial signs and symptoms, assessed at the study visit, and overall disease activity, assessed for the most recent years. The findings were perhaps not unexpected, as the TMJ is a joint among others and a high disease activity affects articular as well as periarticular structures.

The radiographs mirror previous arthritis in contrast with instant clinical and subjective examinations. Panoramic radiography has obvious shortcomings in TMJ diagnostics. Only condylar changes can be evaluated with any confidence, and have acceptable reliability and specificity, but low sensitivity compared with more advanced radiological techniques [40]. For example, joint fluid and synovial enhancement can not be demonstrated with panoramic radiography. Magnetic resonance imaging (MRI) is the most superior technique to detect ongoing inflammation [12,15]. Orofacial signs and symptoms might be related to ongoing inflammation but this is uncertain. However, panoramic examinations are simple, inexpensive, and easily accessible at most clinics, involve low radiation levels and require no sedation in young children. The method is used to get an overview of the jaws and is often performed before more sophisticated imaging examinations are performed. The reasons for the panoramic examinations in our study may have varied and patients with higher disease activity were examined more often, which entails a possible bias. The frequency of condylar alterations in this study was somewhat lower than previously reported for similar patient cohorts [9,10], and were not related to any clinical or subjective variables, ILAR category or disease activity over the last years. Pedersen et al. [10], on the other hand, reported that condylar resorption had the highest prevalence in children with JIA of the polyarticular type as judged from panoramic radiographs. The median disease duration in their study was 4.4 years. Also using panoramic radiographs in patients with JIA, Billau et al. [9] found no relation between condylar lesions, diagnostic category or disease activity after a median disease duration of 2.96 years in agreement with our results.

## Conclusion

The need for prospective studies to find risk factors for TMJ involvement have been emphasized [12] and the findings of this study support that conclusion.

## Competing interests

The authors declare that they have no competing interests.

## Authors’ contributions

All authors have made substantial contributions to and approved the final manuscript.
